# Observations on Medical Reform

**Published:** 1806-06

**Authors:** 


### *To the Editors of the Medical and Physical Journal.*

Gentlemen,

UPON reading a paper signed Veritas in your Journal for March, I was inclined to make the following remarks, which, should you think worthy of a place in your valuable publication, I shall consider myself obliged by your inserting them the first opportunity.

It is not the intention of these remarks to call into question the talents and ingenuity of the author of that paper upon which these lines were written, but to explain some sentences in it, which appear to me likely to be misunderstood, with respect to the intentions, the Medical Reform is expected to answer.

Your Correspondent asks the question, "Is it just that every medical man is to be taxed because such is the folly of public opinion?" Now, if I recollect, taxation was no where recommended as a means whereby the profession would be established upon a more respectable basis; but that every person intending to practice as surgeon-apothecary should undergo an examination, prior to such practice; to include those in the country as well as the metropolis, and all such as were not approved to be subject to similar restrictions and penalties as gentlemen of the law. That the latter has attracted the attention of the legislature we have ample proof; how is it then that the former is passed by unnoticed? Is it that the property of his Majesty's subjects is of more importance than their health and lives? or that attornies are altogether men of science and respectability, whilst surgeons and apothecaries caries are formed from men of learning, to the low gradation of blacksmiths, barbers, coblers, &c. &c. or rather the characters enumerated, without distinction, are suffered to exercise with impunity some part or other of a profession, which, above all others, in my estimation, ought to be confined to those whose training has been equal to the important office of having the health and comforts of their fellow creatures entrusted to their care.

I cannot suppose the "College would sanction any proceeding which would lead to an increase of burthens on the profession," but if such proceedings should meet with their support, and be carried into effect, the advantages would be numerous both to the profession and public at large; it would induce parents to be more careful in the education of such sons as are designed for the profession, and thereby furnish an alternative to the necessity of trusting the compounding of medicines to servile shopmen, &c. &c. It would also stimulate young men to industry in the acquirement of the rudiments of the profession, zealous of being qualified for their examination; the refusal of a diploma being universally received as the greatest mark of disapprobation and opprobium.

There is no branch of the profession to which greater mischief accrues, than the reduction of bones; and as your Correspondent, from his situation, may not have had opportunity of seeing the distressing circumstances which too frequently occur from the practice of bone-setters, I shall take the liberty of submitting a case or two to his perusal, one of which I was consulted in, the other happened within the circle of my medical acquaintance.

The rude manner in which the cases are drawn up, have great need of apology: but an implicit confidence if every surgeon who has witnessed similar baneful effects, would lay them before the public, through the medium of your widely circulated Journal, the reform would, ere long, meet with universal approbation, and be established upon such conditions as would secure a recommendation adequate to our experience, attached to far more information, liberality, and esteem; with these views I am persuaded to publish the following cases.

W. S. about forty years of age, of good habit of body, had the misfortune to fracture his thigh; a surgeon was sent for, who reduced the bone, and the case went on very well for near a fortnight, when some of his friends calling to see him, persuaded him that surgeons knew nothing of bone-setting, recommended him to send for a "bone-

"bone-setter." It was done; the fellow came, who confirmed their opinion, by using so much violence as brought on a very severe fever, that the man's life was despaired of. Another person was then called in, of the fellows naming; and lastly, a physician. To the last gentleman's skill, we very fairly may attribute the preservation of the man's life; but the management of fractures not coming within the province of a physician, after the fever became subdued, the Doctor relinquished his attendance. The man was then removed to the famous Whitworth Doctors, where he remained for near a twelvemonth, before he was able to go by the help of crutches.

G. G. about forty-two years old, dislocated the astragalus; almost the whole of the bone protruded without the integuments. A bonesetter was sent for, who after applying boiling salve to the wound, bound the limb tight up, assuring him of a certain and speedy cure: in rather less than a week the pain and fever (as might be expected from such rash treatment) became so alarming, that a surgeon of the first eminence was requested to visit him; the limb had then a most shocking appearance, and from the acquired habits, &c. of the patient, it was no easy matter to determine upon what plan to adopt; however, it was thought better to remove the dislocated bone, as striking at the root of irritation, and give the only, but trifling chance of preserving the limb, than amputate, the fever and state of the parts affording but small encouragement of success from the last mentioned operation. The man recovered, after a very long confinement; and it will only be justice to attribute it to the correct and judicious management of his surgeon.

Mr. R. having to come over a moor in a dark night, on his way home, fell into a stone quarry, and pitched on the shoulder; the deltoid muscle was so much bruised that he was unable to raise his arm; the next morning he consulted me, when, upon recommending an antiphlogistic diet, with a sedative application to the part, the swelling of the muscular substance began to subside in two or three days; but still being unable to raise his arm without pain, and his avocations requiring his occasional attendance in most of the market towns in this county, he consulted a surgeon of indubitable abilities; interrogating him, as he had done me before, if he did not suppose his shoulder out? The gentleman assuring him it was not, ordered an embrocation, which he used a few days, but not finding the motion of his arm so perfect as it was befoer the accident, nor entirely free from pain, he went to the Whitworth Doctors, who confirmed the idea he had so anxiously cherished, by twisting and pulling him in every direction, applying a plaster and binding it up. The gentleman's whim thus became satisfied, until getting wet on a rainy day, the rheumatism was produced, which was the cause of my patient's return to me, fully convinced of his folly, in subjecting himself to such violent corporeal punishment for the reduction of a dislocated bone, which was perfectly imaginary.

I am, &c.

*May* 1, 1806. W. E.

